# Crosstalk Between Bile Acids and Intestinal Epithelium: Multidimensional Roles of Farnesoid X Receptor and Takeda G Protein Receptor 5

**DOI:** 10.3390/ijms26094240

**Published:** 2025-04-29

**Authors:** Xiulian Lin, Li Xia, Yuanjiao Zhou, Jingchen Xie, Qinhui Tuo, Limei Lin, Duanfang Liao

**Affiliations:** Key Laboratory for Quality Evaluation of Bulk Herbs of Hunan Province, School of Pharmacy, Hunan University of Chinese Medicine, Changsha 410208, China; 20232047@stu.hnucm.edu.cn (X.L.); 15376155784@163.com (L.X.); 202009030248@stu.hnucm.edu.cn (Y.Z.); 004108@hnucm.edu.cn (J.X.); qinhuituo@hnucm.edu.cn (Q.T.); limei_lin@hnucm.edu.cn (L.L.)

**Keywords:** bile acids, bile acid receptors, intestinal epithelial cells, energy metabolism, intestinal microbiota

## Abstract

Bile acids and their corresponding intestinal epithelial receptors, the farnesoid X receptor (FXR), the G protein-coupled bile acid receptor (TGR5), play crucial roles in the physiological and pathological processes of intestinal epithelial cells. These acids and receptors are involved in the regulation of intestinal absorption, signal transduction, cellular proliferation and repair, cellular senescence, energy metabolism, and the modulation of gut microbiota. A comprehensive literature search was conducted using PubMed, employing keywords such as bile acid, bile acid receptor, FXR (nr1h4), TGR5 (gpbar1), intestinal epithelial cells, proliferation, differentiation, senescence, energy metabolism, gut microbiota, inflammatory bowel disease (IBD), colorectal cancer (CRC), and irritable bowel syndrome (IBS), with a focus on publications available in English. This review examines the diverse effects of bile acid signaling and bile receptor pathways on the proliferation, differentiation, senescence, and energy metabolism of intestinal epithelial cells. Additionally, it explores the interactions between bile acids, their receptors, and the microbiota, as well as the implications of these interactions for host health, particularly in relation to prevalent intestinal diseases. Finally, the review highlights the importance of developing highly specific ligands for FXR and TGR5 receptors in the context of metabolic and intestinal disorders.

## 1. Introduction

### 1.1. Physiological Functions of Bile Acids

Bile acids are steroid compounds synthesized through cholesterol metabolism and are integral to various physiological processes in humans [1]. Traditionally, the primary function attributed to bile acids has been their role in facilitating the digestion and absorption of lipids, due to their zwitterionic nature [2]. However, emerging research indicates that bile acids possess a broader and more intricate array of physiological functions [3,4,5,6,7]. Firstly, bile acids enhance the emulsification, digestion, and absorption of fat-soluble vitamins and lipids by forming micelles [8]. This mechanism is essential not only for nutrient absorption but also for the maintenance of cholesterol homeostasis [9]. Secondly, bile acids function as signaling molecules that regulate their own synthesis, transport, and metabolism through the activation of specific nuclear receptors, such as farnesoid X receptor (FXR), and membrane receptors, such as TGR5 [2,10]. Furthermore, bile acids are pivotal in the regulation of glucose and lipid metabolism. Research has demonstrated that bile acids influence liver glycogen synthesis, gluconeogenesis, and fatty acid oxidation via the FXR and TGR5 signaling pathways [11,12]. Bile acids were shown to stimulate enteroendocrine cells, leading to the secretion of intestinal hormones such as glucagon-like peptide-1 (GLP-1), thereby further influencing systemic metabolism [13]. Additionally, bile acids are crucial in maintaining intestinal barrier integrity and modulating intestinal immune responses [14]. They contribute to the preservation of intestinal epithelial integrity by modulating the expression and function of tight junction proteins [15]. Furthermore, bile acids are involved in regulating the composition and metabolic activities of the gut microbiota, thereby influencing host–microbe interactions [16,17]. Recent research elucidated the role of bile acids in regulating cellular processes such as proliferation, differentiation, and apoptosis [18]. These insights enhance our understanding of the physiological functions of bile acids as well as identify potential therapeutic targets for various diseases, including inflammatory bowel disease, metabolic syndrome, and certain cancers [11,14].

### 1.2. Importance of Intestinal Epithelial Cells

Enterocytes, as the principal constituents of the largest mucosal surface within the digestive tract, are crucial for sustaining intestinal functionality and overall systemic health [19]. These highly specialized cells establish a dynamic monolayer barrier that is essential for the selective absorption of nutrients also for defending against pathogenic invasions and preserving intestinal homeostasis [20]. The polarized structure and specialized membrane transporters of enterocytes facilitate the efficient uptake of nutrients [21]. These cells express a diverse array of specific transporters, including glucose transporters SGLT1 and GLUT2, amino acid transporters [22], and fatty acid transporters [23], thereby ensuring the effective absorption of carbohydrates, proteins, and lipids [24]. Furthermore, enterocytes are integral to the intestinal barrier, forming tight junctions, adherens junctions, and desmosome junctions to create a selectively permeable physical barrier [25]. Research has demonstrated that the expression and function of tight junction proteins, including claudins, occludin, and zonula occludens (ZO) proteins, are crucial for preserving the integrity of the intestinal barrier [26]. Furthermore, enterocytes contribute to innate immune defense by producing a range of antimicrobial peptides, such as defensins and cathelicidins, which establish a chemical barrier against pathogens [27]. Concurrently, these cells are capable of recognizing pathogen-associated molecular patterns (PAMPs) and initiating immune responses via pattern recognition receptors, including Toll-like receptors (TLRs) and NOD-like receptors (NLRs) [28,29].

In summary, intestinal epithelial cells are crucial for nutrient absorption, barrier defense, immune regulation, and the maintenance of microbial equilibrium through their diverse functions.

### 1.3. Bile Acids and Bile Acid Receptors

As the terminal products of cholesterol metabolism, bile acids are not only essential for lipid digestion and absorption but also serve as significant signaling molecules that regulate a variety of physiological processes. In humans, primary bile acids (such as cholic acid and chenodeoxycholic acid) are synthesized by the liver, whereas secondary bile acids (such as deoxycholic acid and lithocholic acid) are generated by the intestinal microbiota through the metabolism of primary bile acids. These bile acids exist either in a free state or conjugated with glycine or taurine, forming a complex bile acid pool within the body. The bile acid pool and its receptors constitute an intricate and sophisticated regulatory network, playing a pivotal role in maintaining homeostasis.

Bile acid receptors primarily encompass the farnesoid X receptor (FXR), the vitamin D receptor (VDR), the pregnane X receptor (PXR), constitutive androstane receptor (CAR), the G protein-coupled bile acid receptor (TGR5), sphingosine-1-phosphate receptor 2 (S1PR2), Mas-related G protein-coupled receptor X4 (MRGPRX4), etc. [30]. The expression patterns of these receptors differ across various intestinal segments and cell types, contributing to a complex signaling network. Bile acids and their receptors are integral in regulating intestinal absorption, signal transduction, cell proliferation and repair, cellular senescence, immune responses, energy metabolism, and the microbiota within intestinal epithelial cells. Among these, FXR and TGR5 are the predominant receptors expressed in intestinal epithelial cells. They are key regulatory factors in bile acid metabolism, innate immunity, energy metabolism, and inflammatory response. Therefore, this review will examine the diverse effects of bile acids and these two receptors on epithelial cells.

## 2. Effects of Bile Acids and Bile Acid Receptors on Intestinal Epithelial Cell (IEC) Proliferation

Bile acids and their receptors play a multifaceted and significant role in modulating the proliferation of intestinal epithelial cells. This regulatory effect is both concentration-dependent and receptor-specific, and it is crucial for maintaining the integrity and function of the intestinal epithelium [31]. Bile acids have the capacity to induce proliferation of intestinal epithelial cells while limiting apoptosis [18].

### 2.1. FXR-Mediated Proliferation Regulation

As the principal nuclear receptor for bile acids, FXR is integral to the regulation of IEC proliferation. The regulation of proliferation mediated by FXR encompasses a variety of molecular mechanisms and signaling pathways, with its role exhibiting complexity that is specific to cell type and contingent upon environmental factors.

FXR modulates the proliferation of intestinal epithelial cells through direct influence on the expression of genes associated with the cell cycle [32,33]. Specifically, FXR upregulates cell cycle inhibitors, notably increasing the expression of p21 (CDKN1A) [33,34], which subsequently inhibits epithelial cell proliferation. Empirical evidence indicated that FXR can directly bind to the FXR response element (FXRE) within the promoter region of the p21 gene, thereby enhancing its transcription [35]. Additionally, in certain cell types, FXR is capable of upregulating the expression of p16 (CDKN2A) [36]. The protein p16 functions by inhibiting CDK4/6 activity, thus preventing the transition of cells from the G1 phase to the S phase, effectively regulating cell proliferation [37]. Moreover, FXR activation triggers a pro-apoptotic program in both differentiated normal colon epithelium and transformed colonocytes [33].

**Figure 1:**
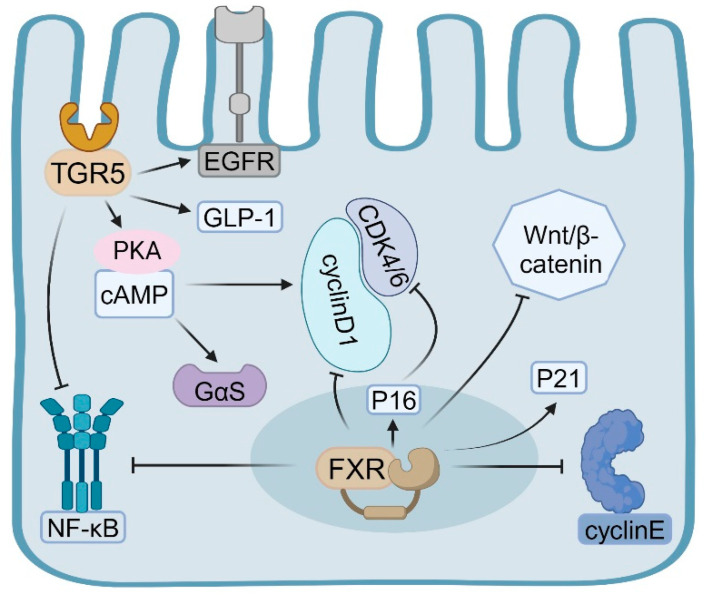
The influence of bile acids and their associated intestinal epithelial receptors, FXR and TGR5, on the proliferation of intestinal epithelial cells.

FXR also exerts regulatory control over cell-cycle-promoting factors. It influences the expression of cyclin D1 [38,39], although its effects are contingent upon the cell type and environmental context. In certain scenarios, FXR activation may suppress cyclin D1 expression, thereby inhibiting the G1/S phase transition [39]. Furthermore, FXR impacts cell cycle progression by modulating the expression of other cell cycle proteins, such as cyclin E [40] (Figure 1). There is a complex interaction between FXR and the Wnt/β-catenin signaling pathway, which is crucial for the regulation of IEC proliferation [41]. FXR activation can inhibit the nuclear translocation of β-catenin, thereby weakening the activity of the Wnt signaling pathway [42]. This inhibitory effect is achieved by increasing the phosphorylation and degradation of β-catenin [43]. FXR can affect the expression of multiple Wnt target genes, such as c-Myc and cyclinD1, thereby regulating cell proliferation [44,45]. Studies have shown that FXR can directly interact with TCF4 (a key transcription factor in the Wnt signaling pathway) and affect its transcriptional activity [41,43,46,47] (Figure 1).

The FXR plays a crucial role in modulating the self-renewal and differentiation of intestinal epithelial stem cells, thereby indirectly influencing the proliferation dynamics of the entire intestinal epithelium [48]. Research has demonstrated that FXR, inherent to intestinal macrophages, detects abnormal bile acids, resulting in the secretion of proinflammatory cytokines that subsequently promote the proliferation of intestinal stem cells. Mechanistically, the activation of FXR ameliorates intestinal inflammation and inhibits tumor growth associated with colitis by modulating the recruitment, polarization, and interaction of intestinal macrophages with Th17 cells. Conversely, the absence of FXR in bone marrow or intestinal macrophages exacerbates intestinal inflammation [49]. FXR influences the maintenance and function of stem cells by regulating the expression of stem cell markers such as Lgr5 and Olfm4 [50,51]. Additionally, FXR activation indirectly affects the maintenance of the stem cell niche by modulating the function of Paneth cells [18,52].

### 2.2. Role of TGR5 in the Cell Cycle

Activation of TGR5 has been shown to facilitate the activation of the EGFR signaling pathway, consequently stimulating cellular proliferation [53]. Research indicates that TGR5 signaling originates from plasma membrane rafts, which enhance EGFR interaction and transcriptional activation. In certain instances, TGR5 can advance cell cycle progression via the cAMP-PKA pathway [54]. Through this pathway, TGR5 modulates the expression of cell cycle proteins, such as Cyclin D1, promoting the transition of cells from the G1 phase to the S phase, thereby accelerating cellular proliferation [55]. Additionally, TGR5 activation can augment cellular resistance to oxidative stress and inflammatory damage, indirectly supporting cell proliferation. Bile acids have been observed to enhance IEC proliferation and mitigate mucosal damage by upregulating TGR5 expression [56,57].

In contrast to the nuclear receptor FXR, TGR5 primarily influences the cell cycle by activating intracellular second messenger systems and downstream signaling pathways. Specifically, TGR5-mediated activation of the cAMP-PKA signaling pathway predominantly stimulates adenylate cyclase through the Gαs protein, resulting in elevated intracellular cAMP levels [58]. Elevated levels of cyclic adenosine monophosphate (cAMP) activate protein kinase A (PKA), which subsequently phosphorylates various downstream target proteins, including those involved in cell cycle regulation [59,60]. PKA is capable of phosphorylating and activating the cAMP-response-element-binding protein (CREB), a crucial transcription factor that modulates the expression of numerous genes associated with the cell cycle [61] (Figure 1).

Activation of the TGR5 receptor facilitates the G1/S phase transition through multiple mechanisms, thereby promoting cellular proliferation [55]. Studies have shown that TGR5 activation upregulates the expression of cyclin D1, a pivotal regulator of the G1/S phase transition [55,62]. Furthermore, TGR5 activation results in the phosphorylation of the epidermal growth factor receptor (EGFR) transmembrane domain, leading to the activation of EGFR and its downstream signaling pathways [63]. Through EGFR transactivation, TGR5 can initiate the MAPK/ERK signaling cascade, which is integral to cell proliferation and survival [61] (Figure 1).

TGR5 indirectly affects cell cycle progression by affecting cellular energy metabolism. TGR5 activation can enhance mitochondrial function and ATP production, providing the necessary energy support for cell cycle progression [64]. Studies have shown that TGR5 reduces ROS generation by inhibiting the NF-κB pathway and activating Nrf2/HO-1 signaling, promoting the expression of antioxidant enzymes and thus protecting against bile duct ligation-induced cholestatic liver disease [65]. Therefore, TGR5 affects cell cycle progression and cell lifespan by regulating cellular antioxidant capacity.

TGR5 exerts an indirect influence on cell cycle progression through its modulation of cellular energy metabolism. Activation of TGR5 has been shown to enhance mitochondrial function and ATP production, thereby supplying the requisite energy for cell cycle advancement [64]. Research indicates that TGR5 mitigates reactive oxygen species (ROS) generation by inhibiting the NF-κB signaling pathway and activating the Nrf2/HO-1 pathway, which promotes the expression of antioxidant enzymes and offers protection against cholestatic liver disease induced by bile duct ligation [65]. Consequently, TGR5 plays a critical role in regulating cell cycle progression and cellular lifespan by modulating the antioxidant capacity of cells. The function of TGR5 varies across different types of intestinal epithelial cells. In intestinal endocrine cells, such as L cells, TGR5 activation primarily influences cellular function rather than proliferation. Notably, studies have demonstrated that ginsenoside compounds can modulate TGR5 activity in L cells, leading to increased expression of GLP-1 [66]. Additionally, bile acid signaling activates intestinal stem cells and promotes epithelial regeneration via TGR5 [18,67].

In addition to its direct influence on the cell cycle, TGR5 exerts an indirect impact on cell proliferation by modulating inflammation and cell survival. Activation of TGR5 can inhibit the NF-κB signaling pathway, thereby reducing the production of inflammatory mediators and fostering an environment conducive to cell proliferation [68]. In certain instances, TGR5 activation enhances cell survival by upregulating the expression of anti-apoptotic proteins, such as Bcl-2 [60,69]. Under specific pathological conditions, the function of TGR5 shifts, contributing to the onset and progression of disease. The role of TGR5 in inflammatory bowel disease is multifaceted; it predominantly exerts a protective effect [67,70], although it can also exacerbate inflammation in some cases [71]. The involvement of TGR5 in colon cancer remains a subject of debate [72]. Several studies have indicated that the activation of TGR5 may facilitate the progression of colon cancer. For instance, when TGR5 is activated by agonists such as INT-777, ursodeoxycholic acid (UDCA), and taurolithocholic acid (TLCA), varying effects are observed across different cancer cell types [73]. Furthermore, TGR5 activation can trigger several signaling pathways, including protein kinase B (AKT), nuclear factor κB (NF-κB), extracellular signal-regulated kinases (ERK1/2), signal transducer and activator of transcription 3 (STAT3), cyclic adenosine monophosphate (cAMP), and Ras homologous protein, all of which are intricately linked to tumorigenesis and cancer progression [72]. The effect of TGR5 activation is contingent upon the tumor stage and the surrounding microenvironment.

### 2.3. Phasic Regulatory Effect of Bile Acid Concentration on Cell Proliferation

The influence of bile acids on the proliferation of intestinal epithelial cells is evidently concentration-dependent, exhibiting a phasic regulatory effect wherein low concentrations stimulate proliferation, whereas high concentrations inhibit it [74,75]. This phasic effect underscores the complexity of bile acids as signaling molecules and highlights the body’s sophisticated regulatory mechanisms for maintaining intestinal homeostasis.

**Figure 2:**
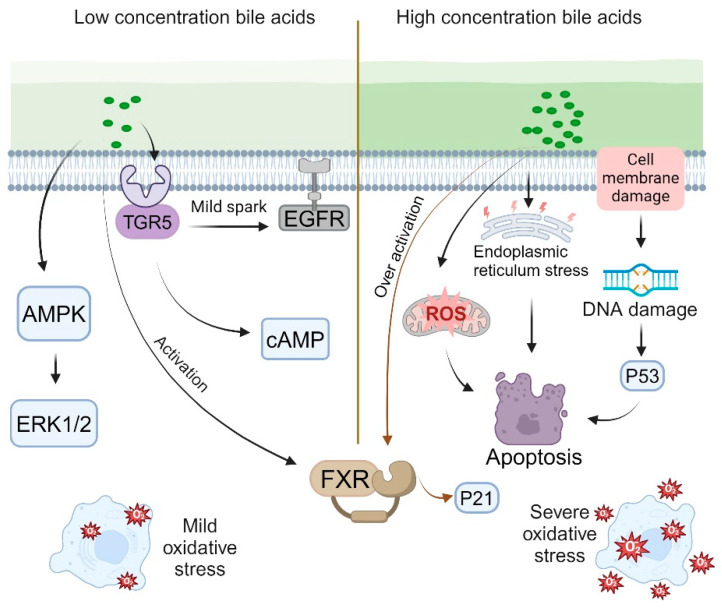
The concentration of bile acids exerts phasic regulatory effects on cellular proliferation.

Within the physiological concentration range, bile acids facilitate the proliferation of intestinal epithelial cells through multiple mechanisms. At low concentrations, bile acids can subtly activate the epidermal growth factor receptor (EGFR) by either promoting ligand release or directly interacting with the receptor [76,77]. Specific bile acids, such as deoxycholic acid and chenodeoxycholic acid (CDCA), are capable of directly or indirectly activating the mitogen-activated protein kinase (MAPK) pathway, particularly the extracellular-signal-regulated kinases 1 and 2 (ERK1/2) [76,78]. Additionally, low concentrations of bile acids can elevate intracellular cyclic adenosine monophosphate (cAMP) levels by activating the TGR5 receptors [54,60]. Furthermore, low concentrations of bile acids induce mild oxidative stress. Research indicates that glycocholic acid (GCA) and glycoursodeoxycholic acid (GUDCA) protect retinal pigment epithelial (RPE) tight junctions from oxidative damage within the concentration range of 100–500 μM, whereas glycodeoxycholic acid (GDCA) offers protection in the range of 10–500 μM. This moderate oxidative stress activates the cell’s protective mechanisms, thereby promoting cell proliferation [79,80] (Figure 2, left). Moreover, in certain instances, moderate activation of the FXR indirectly stimulates cell proliferation by modulating the expression of specific genes, such as fibroblast growth factor 19 (FGF19).

When bile acid concentrations surpass a specific threshold, their influence on cellular proliferation shifts to an inhibitory effect [81]. Elevated bile acid levels can directly inflict damage on cell membranes and DNA [82]. Such DNA damage may activate the p53 pathway, resulting in cell cycle arrest or apoptosis [83]. Additionally, high bile acid concentrations markedly increase the production of reactive oxygen species (ROS), culminating in severe oxidative stress [83,84]. This excessive oxidative stress can impair organelle function and trigger apoptotic pathways [83,85]. Furthermore, elevated bile acid levels induce endoplasmic reticulum (ER) stress and activate the unfolded protein response (UPR) [86,87,88]. Prolonged ER stress may also result in cell cycle arrest and apoptosis [89]. Mitochondrial dysfunction, another consequence of high bile acid concentrations, can lead to disruptions in energy metabolism and cell death [86,90]. Moreover, excessive bile acid concentrations excessively activate the FXR and upregulate the expression of cell cycle inhibitory factors such as p21 [35,91] (Figure 2, right).

The phasic regulatory effect of bile acids on cell proliferation is subject to a concentration threshold that varies according to cell type and the specific bile acid involved [92]. Various IEC types, including absorptive cells, goblet cells, and Paneth cells, exhibit differing sensitivities to bile acids [93,94,95]. Absorptive cells demonstrate heightened sensitivity to bile acids via specific transporters, such as OATP and ASBT [96,97]. Goblet cells play a crucial role in secreting mucus to establish a protective barrier within the intestine, and the presence of bile acids enhances mucus secretion, thereby safeguarding the intestinal epithelium from bile acid stimulation [98]. Research indicates that in rats subjected to a high-fat diet, there is an increase in intestinal bile acid secretion, which leads to the upregulation of the TGR5 in Paneth cells, consequently promoting the proliferation of intestinal epithelial cells [95]. Furthermore, intestinal epithelial stem cells exhibit particular sensitivity to fluctuations in bile acid concentrations [67]. Various bile acids, such as cholic acid, deoxycholic acid, and lithocholic acid, exhibit distinct concentration thresholds and exert differential effects [99,100,101] (Figure 2).

The phasic regulatory influence of bile acid concentrations on cellular proliferation holds significant physiological and pathological implications. This dual regulation is crucial for maintaining the normal renewal rate of intestinal epithelial cells, ensuring a balance between cell proliferation and shedding. Moreover, bile acids play a pivotal role in preserving intestinal health by modulating the proliferation of intestinal stem cells through the activation of the intestinal FXR [50]. Several research data have shown that fluctuations in bile acid concentrations are intricately linked to the proliferation and differentiation of intestinal stem cells, with elevated bile acid levels potentially increasing the risk of intestinal cancer [67]. Furthermore, bile acids influence the proliferation of intestinal epithelial cells by modulating the cell cycle and mitochondrial biogenesis [31].

Following intestinal injury, bile acids are released and facilitate the regeneration of the intestinal epithelium, primarily through the activation of the bile acid receptor TGR5 [56]. Concurrently, bile acids contribute to epithelial renewal by modulating intracellular energy metabolism [102]. Nevertheless, an excessive accumulation of bile acids can impede the proliferation of intestinal stem cells, thereby disrupting intestinal homeostasis [103]. Consequently, the dual regulatory role of bile acids within the intestine is crucial for sustaining the normal renewal rate of the intestinal epithelium. By maintaining optimal bile acid concentrations, a balance between cellular proliferation and homeostasis can be achieved, thereby enhancing intestinal health [31,103,104].

## 3. Role of Bile Acids and Bile Acid Receptors in Intestinal Epithelial Cell (IEC) Differentiation

### 3.1. Effect of FXR on Cell Fate Determination

**Figure 3:**
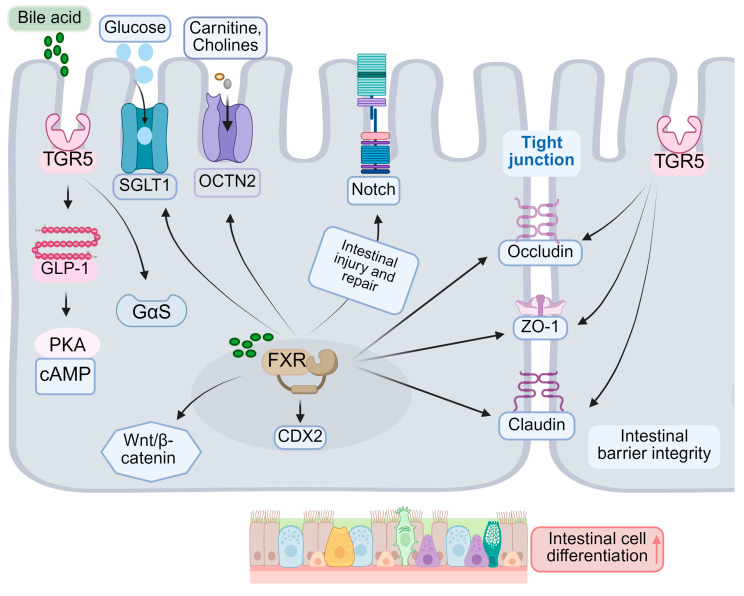
The function of bile acids and bile acid receptors in the differentiation of intestinal epithelial cells.

As the primary bile acid nuclear receptor, FXR (nuclear receptor 1H4) is integral to the differentiation of intestinal epithelial cells [105]. FXR influences cell fate determination by modulating the expression of genes associated with differentiation, impacting the Notch signaling pathway, and regulating the differentiation of intestinal epithelial stem cells. Specifically, FXR can directly enhance the expression of several differentiation markers, including alkaline phosphatase (ALP) and sucrase-isomaltase (SI) [106,107]. Activation of FXR also leads to increased expression of CDX2, a key regulator of IEC differentiation [108]. Furthermore, research indicates that FXR modulates the Notch signaling pathway [109], which is crucial in determining the fate of absorptive and secretory cells [110]. The Notch signaling pathway is integral to the regulation of homeostasis and differentiation of intestinal stem cells. Notch1 and Notch2 receptors are expressed within the intestinal epithelium, with evidence indicating that Notch1 predominantly regulates the function and proliferation of these stem cells [111] (Figure 3). Disruption of Notch signaling results in a reduction of intestinal stem cells and impairs their regenerative capacity, underscoring its essential role in intestinal repair processes [112]. Furthermore, Notch signaling influences the function of intestinal epithelial stem cells by modulating the proliferation and differentiation of Lgr5+ precursor cells [50]. During the differentiation process of intestinal stem cells, the interplay between the FXR and Notch signaling pathways may influence cell fate decisions. Research suggests that activation of Notch signaling facilitates the differentiation of intestinal stem cells into absorptive cells, whereas FXR activation may modulate this process by regulating the expression of genes associated with differentiation [113]. Therefore, the coordinated interaction between the FXR and Notch signaling pathways plays a crucial role in determining the fate of intestinal stem cells.

FXR contributes to intestinal protection against harmful substances by enhancing the barrier function during the differentiation of intestinal epithelial cells. It regulates the expression of tight junction proteins, such as occludin and claudin [114], thereby strengthening the integrity of the intestinal epithelial barrier and reducing permeability. Although FXR is expressed at low levels in intestinal epithelial stem cells, its activation facilitates the differentiation of these stem cells into mature epithelial cells, partially through the modulation of the Wnt/β-catenin signaling pathway. In vivo, microbial-derived isoDCA enhances the immunostimulatory properties of dendritic cells by inhibiting FXR activity, which indirectly promotes the differentiation of colonic Tregs [18]. FXR is implicated in the maturation and differentiation of innate lymphoid cell (ILC) precursors [115] (Figure 3). Nonetheless, prior research has demonstrated that the nuclear receptor FXR plays a pivotal role in the functional maturation of hepatocyte-like cells (HLCs). Stem cell-derived hepatocytes exhibit a hybrid phenotype, possessing characteristics of both hepatocytes and intestinal cells. Investigations have revealed that FXR inhibits the intestinal traits of HLCs while promoting hepatic characteristics, thereby rendering stem-cell-derived cells more akin to primary hepatocytes [116]. Consequently, FXR facilitates the differentiation of intestinal epithelial cells into mature cells by modulating the expression of specific genes. Activation of FXR can enhance the differentiation of intestinal absorptive cells, such as intestinal epithelial cells, thereby augmenting their nutrient absorption capacity. This effect is typically associated with the upregulation of specific transporters, including the sodium-dependent glucose transporter SGLT1 [117] and the organic cation transporter OCTN2 [118].

### 3.2. Role of TGR5 in Cell Differentiation

While TGR5 is primarily recognized as a receptor involved in the regulation of metabolism and inflammation, recent research has demonstrated its role in the differentiation of intestinal epithelial cells [60]. Activation of TGR5 facilitates the differentiation of L cells and the secretion of GLP-1 [119,120]. In the intestinal environment, bile acids bind to TGR5, activating the Gαs signaling pathway and subsequently increasing intracellular cAMP levels [54,121] (Figure 3). This cascade further stimulates protein kinase A (PKA) and other downstream signaling pathways, thereby influencing cell differentiation. The activation of intestinal TGR5 enhances the expression of various differentiation markers, including intestinal-specific proteins such as MUC2, OCLN, and ZO-1, which are crucial for maintaining intestinal barrier function and promoting cell differentiation [122]. Moreover, studies indicate that TGR5 activation stimulates the proliferation and differentiation of intestinal epithelial cells as well as strengthens the integrity of the intestinal barrier by modulating the expression of tight junction proteins [123,124,125]. For instance, MUC2, the primary protein constituent of intestinal mucus, serves a protective role for the intestinal epithelium against pathogenic invasion, whereas OCLN and ZO-1 are critical components of tight junctions, essential for maintaining intercellular barrier integrity [126]. Furthermore, the activation of TGR5 is implicated in the remodeling of the intestinal microbiota, thereby supporting the preservation of intestinal health and immune function [61,127]. Under specific conditions, TGR5 influences the differentiation of enterochromaffin cells [128]. TGR5 contributes to the maintenance of barrier function in mature intestinal epithelial cells by enhancing the expression of tight junction proteins [129]. The involvement of TGR5 in intestinal processes presents a novel therapeutic target for a range of intestinal disorders. For example, research indicates that TGR5 activation may have therapeutic potential in the management of IBD, IBS, and other related conditions.

### 3.3. Effects of Bile Acids on Intestinal Epithelial Stem Cell Differentiation

Bile acids influence the functionality of Paneth cells via the activation of the FXR, which encompasses the secretion of antimicrobial peptides and the synthesis of growth factors [130,131]. The growth factors produced by Paneth cells are crucial for providing essential growth signals to intestinal stem cells, thereby facilitating their self-renewal and differentiation [132]. The normal functioning of Paneth cells, characterized by the adequate secretion of growth factors, is advantageous for the stability and maintenance of intestinal stem cell functions [133]. Furthermore, the antimicrobial activity of Paneth cells plays a pivotal role in regulating the intestinal microbiota and mitigating inflammatory responses induced by bacterial infections [134]. Inflammation adversely affects the functionality of intestinal stem cells, impeding their proliferation and differentiation [135,136]. Consequently, the integrity of Paneth cells is essential for sustaining the function of intestinal stem cells. Additionally, bile acids influence the expression and degradation of basement membrane proteins [137], thereby altering the physicochemical properties of the stem cell microenvironment [138,139,140]. Bile acids also indirectly modulate stem cell behavior by regulating the function of intestinal stromal cells, such as myofibroblasts [141,142].

Bile acids modulate β-catenin activity via the FXR, consequently influencing the Wnt signaling pathway, which is crucial for stem cell self-renewal and differentiation regulation [143]. Furthermore, bile acids impact the expression of Notch receptors or ligands, thereby modulating Notch signaling activity. The Wnt signaling pathway is integral to maintaining stem cell self-renewal and differentiation regulation. Research indicates that bile acids influence the Notch signaling pathway by altering the expression of Notch receptors or ligands [144]. The Notch signaling pathway is pivotal in various biological processes, including cell fate determination, tissue regeneration, and tumorigenesis [145,146]. It plays a significant role in liver development and regeneration, and the modulation of bile acids may affect intestinal stem cell function. Studies have demonstrated that the Notch signaling pathway is involved in liver development and regeneration by regulating the proliferation and differentiation of liver stem cells [144]. Furthermore, Notch signaling is essential for maintaining the integrity and functionality of intestinal epithelial cells, particularly in the context of intestinal inflammation and immune homeostasis [147]. Bile acids, as signaling molecules, can influence Notch signaling activity by modulating liver and intestinal metabolic processes, thereby impacting the fate of intestinal stem cells [148]. Alterations in the composition and concentration of bile acids are closely associated with changes in the intestinal microbiota, which may in turn affect the activation state of Notch signaling, influencing the self-renewal and differentiation of intestinal stem cells [149] (Figure 3). Notch signaling is pivotal in determining the fate of absorptive and secretory cells. Additionally, bile acids modulate the intensity of bone morphogenetic protein (BMP) signaling by affecting the expression of BMP ligands or receptors, which are critical for regulating stem cell differentiation and the formation of the crypt–villus axis.

## 4. Relationship Between Bile Acids, Bile Acid Receptors, and Intestinal Epithelial Cell (IEC) Aging

### 4.1. Bile-Acid-Induced Oxidative Stress and DNA Damage

**Figure 4:**
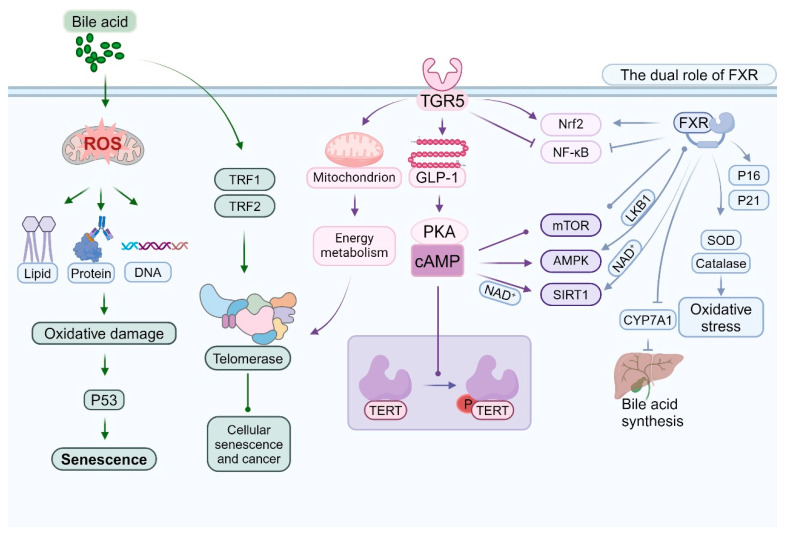
The association between bile acids, bile acid receptors, and the senescence of intestinal epithelial cells.

Bile acids and their receptors play a multifaceted and significant role in the senescence of intestinal epithelial cells. This intricate relationship encompasses various dimensions, including oxidative stress, DNA damage, cell cycle regulation, and metabolic alterations. The aging process is frequently associated with inflammation, dysregulated bile acid (BAS) homeostasis, and intestinal dehydration [150]. Elevated concentrations of specific bile acids, such as deoxycholic acid, can enhance the production of reactive oxygen species (ROS) [151]. Persistent oxidative stress may expedite cellular aging and induce oxidative damage to proteins, lipids, and DNA [152]. Certain secondary bile acids have the potential to cause DNA damage directly or indirectly, including single-strand and double-strand breaks [153]. The accumulation of DNA damage is a hallmark of cellular aging and can activate aging-related pathways, such as p53 [154,155] (Figure 4).

As the principal nuclear receptor for bile acids, FXR exerts intricate regulatory effects during cellular aging. Dysfunction of the intestinal barrier is recognized as an evolutionarily conserved hallmark of aging [156]. In the context of anti-aging, activation of FXR can enhance the expression of antioxidant genes, including SOD and catalase, thereby mitigating oxidative stress [157,158]. FXR also attenuates the production of inflammatory mediators and alleviates aging-associated chronic inflammation by inhibiting the NF-κB signaling pathway [159]. Research indicates that FXR serves as a target for the prevention of diet- and aging-related metabolic disorders [160]. Our findings indicate that the down-regulation of FXR plays a pivotal role in the development of aging-induced fatty liver [161]. Additionally, FXR influences the cellular aging process by modulating the expression and activity of SIRT1 [162,163]. Regarding its potential role in promoting aging, under specific conditions, prolonged FXR activation induces cellular senescence by upregulating cell cycle inhibitors such as p16 and p21 [35,36,164]. Furthermore, excessive activation of FXR can disrupt cellular metabolism and indirectly expedite the aging process [165] (Figure 4).

TGR5 is critically involved in the regulation of cellular senescence [166]. Activation of TGR5 enhances cellular resistance to oxidative stress, partially through the activation of the Nrf2 signaling pathway [65,167,168]. Furthermore, TGR5 contributes to the maintenance of cellular energy homeostasis and delays the aging process by modulating mitochondrial function [166,169]. It also indirectly influences cellular aging by regulating GLP-1 secretion and energy metabolism. Certain bile acids modulate the mTOR signaling pathway via TGR5, thereby influencing cellular senescence and autophagy [170,171,172]. Recent studies have explored the interactions and potential mechanisms linking ileitis and metabolic-associated steatotic liver disease (MASLD). Changes in intestinal flora induced by MASLD result in elevated levels of secondary bile acids in the ileum. In the context of a compromised intestinal barrier, this leads to severe CD8+ T cell-mediated ileitis through the TGR5/mTOR/OXPHOS signaling pathway. Tissue damage induced by ileitis disrupts enterohepatic circulation, inhibits hepatic FXR activation, and exacerbates the MASLD phenotype [173]. This inflammatory milieu fosters enterocyte senescence by promoting oxidative stress and DNA damage [174,175]. Activation of TGR5 has been shown to influence the AMPK pathway, thereby modulating energy metabolism and cellular senescence [120,176] (Figure 4). 

Activation of TGR5 has been shown to influence the AMPK pathway, thereby modulating energy metabolism and cellular senescence [120,176]. The intestinal microbiome plays a crucial role in bile acid metabolism and the aging of intestinal epithelial cells [16,177]. Specifically, the intestinal flora is capable of converting primary bile acids into secondary bile acids, which are more likely to promote cellular aging [150,178]. With advancing age, alterations in intestinal microbial diversity impact bile acid composition, subsequently influencing cellular aging processes [150,179].

Dysregulation of bile acid metabolism is linked to a range of aging-related gastrointestinal diseases [180]. Additionally, aging modifies bile acid metabolism and receptor functionality, thereby increasing the susceptibility to inflammatory bowel disease [15,150,172]. Prolonged exposure to elevated concentrations of certain bile acids has been associated with the development of colon cancer, a condition that correlates with cellular aging and the accumulation of DNA damage [181]. Consequently, anti-aging strategies that leverage bile acid signaling and elucidate the roles of bile acids and their receptors in cellular aging may provide a foundation for the development of novel anti-aging interventions.

### 4.2. Relationship Between Bile Acid and Telomerase Activity

Telomerase is a crucial enzyme responsible for maintaining telomere length, thereby playing a pivotal role in cellular aging and the regulation of lifespan. Recent research has demonstrated that bile acids and their receptors influence telomerase activity through various mechanisms, thus contributing to the regulation of cellular lifespan [182]. Specifically, certain bile acids have been shown to directly modulate telomerase activity by impacting the transcription of the telomerase reverse transcriptase (TERT) gene. Studies have shown that bile acids, at specific concentrations, can upregulate TERT expression, potentially enhancing telomerase activity. This observation is significantly linked to the role of telomerase in cellular proliferation and oncogenesis [183,184,185]. In certain cancer types, the reactivation of telomerase is considered a critical factor in tumor progression. For instance, mutations in the TERT promoter are commonly identified in urothelial carcinoma and are linked to increased telomerase activity and enhanced tumor invasiveness [183]. Furthermore, bile acids, such as ursodeoxycholic acid (UDCA), have been demonstrated to inhibit the proliferation of colorectal cancer cells by modulating the YAP signaling pathway, potentially through their impact on telomerase activity [184]. In hepatocellular carcinoma, alterations in bile acid concentrations are similarly associated with telomerase reactivation and increased tumor invasiveness [185].

The mechanism by which bile acids exert their effects may involve the regulation of TERT transcription, complementing other established regulatory mechanisms. Notably, TERT expression is influenced not only by transcription factors but also by epigenetic modifications. Recent research has indicated that the transcriptional activity of TERT is closely associated with the methylation status of its promoter region, particularly in cancer cells [186]. In thyroid cancer, TERT overexpression is linked to promoter mutations and epigenetic alterations, which collectively influence TERT transcriptional activity [187]. Furthermore, research has demonstrated that template activation factor I (TAF-I) plays a role in regulating TERT transcription by maintaining histone modifications and demethylated cytosine, both of which are associated with transcriptional activation [188]. These findings imply that bile acids may further facilitate cancer cell proliferation and survival by influencing the transcriptional regulatory network of TERT [189]. Additionally, studies have indicated that the atypical functions of TERT within cells may also contribute to its involvement in cancer, particularly through its roles in gene expression regulation and cell proliferation [190,191]. The identification of these non-canonical functions offers a more comprehensive perspective, enhancing our understanding of the multifaceted roles of TERT in cell biology.

The activation of the FXR indirectly modulates the expression of telomerase reverse transcriptase (TERT), primarily by influencing the activity of specific transcription factors. As a nuclear receptor, FXR is implicated in the regulation of various metabolic processes, including those related to bile acid, lipid, and glucose metabolism [192]. Scholars have conducted research indicating that FXR activation impacts hepatic and intestinal metabolism also plays a crucial role in cellular proliferation and survival [193]. Furthermore, FXR indirectly influences telomerase activity and assembly by modulating genes associated with the cell cycle and proliferation [194]. Recent research has highlighted the significance of the TGR5 in numerous physiological and pathological processes, such as metabolic diseases, inflammatory responses, and hepatic disorders [195]. Activation of TGR5 indirectly affects the phosphorylation and activity of TERT via the cAMP-PKA signaling pathway [68,196]. Additionally, TGR5 can modulate telomerase activity by altering the cellular energy metabolism state [195,197] (Figure 4).

Research has demonstrated that telomerase activity is modulated by various signaling pathways, with histone modification playing a pivotal role in this regulation [198]. Furthermore, while the chromatin structure of telomeres is traditionally classified as heterochromatin, recent investigations have revealed that telomeres in certain plant models may exhibit characteristics of true chromatin. This finding implies the existence of distinct regulatory mechanisms across different organisms [199]. High-resolution studies of telomerase structure enhance our comprehension of its interactions with substrates and pinpoint mutations that influence its activity [200]. Such structural insights are vital for the development of therapeutics aimed at effectively modulating telomerase activity, particularly in the context of diseases like cancer. Consequently, the impact of bile acids on the chromatin structure of the telomere region could further influence cell proliferation and tumor progression by modulating the accessibility and activity of telomerase.

Bile acids influence the expression and functionality of specific telomere-binding proteins, including TRF1 and TRF2. Alterations in these proteins subsequently impact the interaction between telomerase and telomeres. TRF1 and TRF2 are integral components of the telomere protection complex, known as shelterin, and are crucial for maintaining telomere length and safeguarding chromosome ends [201]. Research indicates that the binding affinity of TRF1 and TRF2 is modulated by nucleosome organization, which may further influence their functional roles at telomeres [202] (Figure 4). Moreover, TRF2 is significant in the differentiation and maintenance of neural progenitor cells, underscoring its multifaceted roles in cell fate determination [203]. Consequently, bile acids may indirectly modulate telomerase activity and telomere stability by regulating the expression and function of these telomere-binding proteins, thereby contributing to biological processes such as cellular aging and oncogenesis.

### 4.3. Interaction Between Bile Acid Receptors and Aging-Related Signaling Pathways (e.g., mTOR, AMPK, SIRT1)

Bile acid receptors, such as the FXR and the TGR5, engage in intricate interactions with several critical signaling pathways associated with aging. These interactions are pivotal in modulating cellular metabolism, stress responses, and lifespan regulation [7,204]. Activation of FXR influences the mechanistic target of rapamycin complex 1 (mTORC1) through various mechanisms [205,206,207], while TGR5 activation modulates mTOR activity via the cAMP-PKA signaling pathway [60,208]. This modulation is crucial for the regulation of autophagy and protein synthesis [209,210]. Alterations in mTOR activity subsequently impact the expression of genes involved in bile acid synthesis and transport [211], establishing a complex feedback loop between bile acid signaling and mTOR regulation [212] (Figure 4).

The activation of TGR5 indirectly stimulates AMPK by elevating intracellular cAMP levels [213,214]. The activation of AMPK constitutes a crucial mechanism through which TGR5 modulates energy metabolism [215]. FXR indirectly influences AMPK activation by modulating the expression or activity of LKB1 [216]. Subsequently, AMPK activation impacts the transcriptional activity of FXR [217]. AMPK further influences bile acid synthesis by regulating the expression of key enzymes, such as CYP7A1 [218]. This regulation establishes an additional feedback loop within the bile acid–AMPK signaling network [219] (Figure 4). FXR also indirectly modulates SIRT1 activity by affecting NAD+ metabolism [220]. SIRT1, in turn, regulates its transcriptional activity through the deacetylation of FXR [221]. TGR5 activation indirectly modulates SIRT1 activity by influencing mitochondrial function and NAD+ levels [222]. This interaction plays a significant role in the regulation of energy metabolism and anti-aging processes. Furthermore, SIRT1 affects the expression of genes associated with bile acid metabolism by modulating the activity of FXR and other transcription factors [223] (Figure 4).

Bile acid receptors are pivotal in cellular metabolic reprogramming through their interactions with mTOR, AMPK, and SIRT1 [224,225]. This integration influences cellular responses to nutritional and stress stimuli, thereby impacting the aging process [160]. FXR and TGR5 contribute to the regulation of autophagy by modulating the activities of mTOR and AMPK [173,226]. Precise regulation of autophagy is crucial for maintaining cellular homeostasis and mitigating the effects of aging [227]. Furthermore, the interaction of bile acid receptors with AMPK and SIRT1 plays a significant role in the regulation of mitochondrial biogenesis and function, which is essential for sustaining cellular energy balance and delaying the aging process. The interplay between bile acid receptors and key molecular targets, including mTOR, AMPK, and SIRT1, is integral to the pathogenesis of neurodegenerative disorders, particularly Alzheimer’s disease [228,229]. Furthermore, the interactions between bile acid receptors and aging-related signaling pathways significantly influence vascular function and cardiac metabolism, thereby impacting the aging process of the cardiovascular system [230,231]. Consequently, future therapeutic strategies are proposed to concurrently target bile acid receptors and critical aging pathways—such as employing mTOR inhibitors and AMPK activators—while integrating personalized treatment approaches and time-dependent interventions to achieve synergistic effects in combating aging.

## 5. Regulation of Energy Metabolism of Intestinal Epithelial Cells by Bile Acids and Bile Acid Receptors

### 5.1. Role of FXR in Lipid and Glucose Metabolism

**Figure 5:**
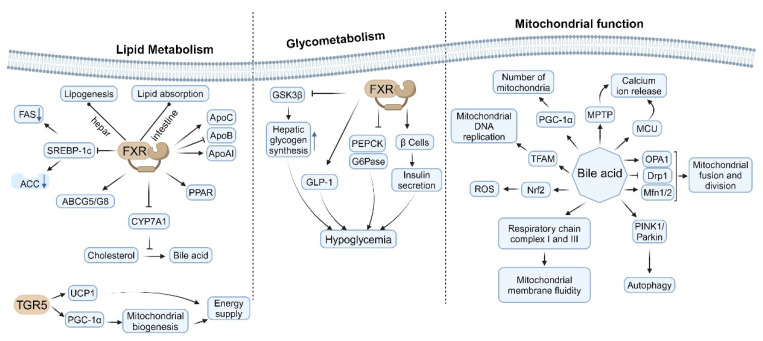
The impact of bile acids and their corresponding receptors on glucose and lipid metabolism, as well as mitochondrial function.

The farnesoid X receptor (FXR), a nuclear receptor activated by bile acids, is integral not only to the regulation of bile acid metabolism but also to lipid and glucose metabolism [232]. FXR is pivotal in maintaining metabolic homeostasis through the direct regulation of gene expression and the indirect modulation of various metabolic pathways [9,233,234]. It exerts an inhibitory effect on the expression of key enzymes involved in fatty acid synthesis, such as acetyl-CoA carboxylase (ACC) and fatty acid synthase (FAS) [235,236], thereby attenuating fatty acid synthesis. This inhibition is partially mediated by the downregulation of sterol regulatory element-binding protein-1c (SREBP-1c) [158,236,237] (Figure 5, left). Furthermore, research has demonstrated that the FXR-dependent reduction in polyunsaturated fatty acids is facilitated by decreased lipid absorption. Utilizing tissue-specific FXR knockout mice, researchers have shown that hepatic FXR regulates lipogenic genes, whereas intestinal FXR modulates lipid absorption, thereby delineating two distinct pathways through which FXR influences hepatic lipid regulation [238]. Activation of FXR has been shown to decrease triglyceride levels in both plasma and liver [239,240,241]. This phenomenon encompasses the inhibition of triglyceride synthesis and the enhancement of fatty acid oxidation. The FXR modulates the expression of apolipoproteins, including apoC [242], apoB [243], and apoAI [244] (Figure 5, left). Through this regulatory mechanism, FXR influences the composition and metabolism of lipoproteins. Furthermore, FXR indirectly impacts the conversion of cholesterol to bile acids by modulating the expression of CYP7A1 [245,246]. Additionally, FXR governs the enterohepatic circulation of cholesterol by affecting the expression of ABCG5/G8. The expression of FXR in adipose tissue plays a role in adipocyte differentiation and function [240,247,248,249,250], involving the regulation of lipogenesis-related genes within the peroxisome-proliferator-activated receptor (PPAR) family [251,252,253] (Figure 5, left).

The FXR plays a significant role in modulating hepatic glycogen synthesis through the regulation of glycogen synthase kinase 3β (GSK3β) expression [254,255]. Activation of FXR enhances hepatic glycogen storage and exerts inhibitory effects on hepatic lipogenesis and gluconeogenesis, thereby promoting lipid metabolism, glycogen synthesis, and insulin sensitivity, which collectively contribute to the reduction of blood glucose levels [256]. Notably, FXR suppresses the expression of critical gluconeogenic enzymes, including phosphoenolpyruvate carboxykinase (PEPCK) and glucose-6-phosphatase (G6Pase) [236]. This suppression leads to a decrease in hepatic glucose output, thereby facilitating improved glycemic control [257] (Figure 5, middle). Additionally, FXR activation enhances insulin sensitivity through various mechanisms [258], such as reducing hepatic steatosis [259], enhancing lipid metabolism [248,260], and exerting anti-inflammatory effects [261]. Furthermore, FXR expression in pancreatic β cells influences insulin secretion and β cell viability [262] by modulating cellular metabolism and antioxidant defenses. FXR also indirectly impacts glucose metabolism by regulating the secretion of glucagon-like peptide-1 (GLP-1) [263], and it affects intestinal glucose absorption and metabolism [264] (Figure 5, middle).

The activation of FXR mitigates the progression of non-alcoholic fatty liver disease (NAFLD) by enhancing lipid metabolism and diminishing liver inflammation [265,266]. FXR agonists demonstrate potential in augmenting insulin sensitivity and regulating blood glucose levels. Consequently, FXR has emerged as a promising target for the treatment of metabolic syndrome, owing to its multifaceted roles in lipid and glucose metabolism. Notably, FXR and PPARα exhibit synergistic effects in the regulation of fatty acid oxidation and lipid metabolism [267]. In contrast, FXR and LXR exert antagonistic effects in the regulation of lipid and cholesterol metabolism [268,269]. Furthermore, FXR influences insulin signaling by modulating the expression or activity of insulin receptor substrates (IRS) [270].

### 5.2. TGR5-Mediated Increase in Energy Expenditure

Recent research has elucidated that TGR5 plays a pivotal role in regulating energy expenditure, offering a novel perspective on the role of bile acids in metabolic regulation [271,272].

Upon activation, TGR5 stimulates adenylate cyclase via the Gαs protein, resulting in elevated intracellular cyclic adenosine monophosphate (cAMP) levels [58,273]. The increase in cAMP subsequently activates protein kinase A (PKA), which initiates a cascade of downstream effects [60,274]. Research indicates that TGR5 activation also modulates intracellular calcium ion concentrations, a process linked to the regulation of energy expenditure [275]. TGR5 ligands facilitate an increase in intracellular calcium concentrations by promoting calcium influx [276], thereby enhancing β-cell insulin secretion through the modulation of potassium and calcium currents, which affects the activation of acutely promoted stimulus–secretion coupling (SSC) [59].

Furthermore, TGR5 activation significantly upregulates the expression of uncoupling protein 1 (UCP1) in brown adipocytes [273,277]. UCP1 is essential for thermogenesis in brown adipose tissue, and its increased expression directly augments energy expenditure [278]. Additionally, TGR5 activation stimulates mitochondrial biogenesis via the activation of peroxisome-proliferator-activated receptor gamma coactivator 1-alpha (PGC-1α) [279]. The resultant increase in mitochondrial number and function enhances the thermogenic capacity of brown adipose tissue [169]. Moreover, TGR5 activation promotes fatty acid oxidation by influencing the expression or activity of key enzymes involved in this metabolic pathway [170,280] (Figure 5, left).

### 5.3. Effects of Bile Acids on Mitochondrial Function

Bile acids, as critical signaling molecules, play a significant role in lipid digestion and metabolic regulation, while also exerting diverse effects on mitochondrial function. These effects are dose-dependent and vary under physiological and pathological conditions. Certain bile acids can upregulate the expression of PGC-1α by activating TGR5 or FXR receptors [281], with PGC-1α serving as a key regulator of mitochondrial biogenesis, thereby promoting an increase in mitochondrial numbers. Additionally, bile acids influence the expression or activity of proteins involved in mitochondrial DNA replication [31], such as TFAM.

At low concentrations, bile acids enhance the activity of respiratory chain complexes, particularly complexes I and III [282]. Conversely, high concentrations inhibit respiratory chain function, leading to reduced ATP production [283]. Bile acids also modulate the efficiency of electron transfer by altering mitochondrial membrane fluidity [284] (Figure 5, right), with low concentrations proving beneficial and high concentrations detrimental. Physiological concentrations of bile acids play a crucial role in maintaining mitochondrial membrane potential and promoting energy metabolism. In contrast, elevated concentrations of bile acids result in the collapse of membrane potential, thereby inducing apoptosis [285,286] and inhibiting energy metabolism. Bile acids influence the function of ATP synthase (complex V), consequently affecting the proton gradient and membrane potential [287,288].

Furthermore, bile acids modulate calcium uptake by impacting the mitochondrial calcium uniporter (MCU). Certain bile acids influence the opening of the mitochondrial permeability transition pore (MPTP), thereby affecting calcium release [86,289,290] (Figure 5, right). At low concentrations, bile acids decrease reactive oxygen species (ROS) production by upregulating the expression of antioxidant enzymes [31]. Conversely, high concentrations of bile acids enhance ROS production, leading to oxidative damage [84]. Some bile acids can activate the Nrf2 pathway, thereby enhancing mitochondrial antioxidant defense [291,292]. Additionally, bile acids activate Nrf2 in intestinal cells, and the intestinal-cell-specific knockout of Nrf2 increases the susceptibility of fruit flies to bile-acid-induced toxicity [293]. Bile acids influence mitochondrial fusion and fission by modulating the expression or activity of proteins such as Mitofusin 1/2 (Mfn1/2) [294], dynamin-related protein 1 (Drp1) [295], and optic atrophy 1 (OPA1) [290]. Additionally, bile acids regulate the process of mitochondrial autophagy through their impact on the PINK1/Parkin pathway [295,296] (Figure 5, right).

### 5.4. Interaction Between Bile Acid Receptors and Metabolism-Related Hormones (Such as GLP-1)

The interactions between bile acid receptors and metabolism-related hormones are complex, as exemplified by glucagon-like peptide-1 (GLP-1). Bile acid receptors, particularly the FXR and the TGR5, engage in intricate interactions with various metabolism-related hormones. These interactions are crucial for maintaining overall metabolic homeostasis, with the interaction involving GLP-1 being especially significant.

The activation of TGR5 in intestinal L cells significantly enhances the secretion of GLP-1 [297,298], thereby facilitating improvements in glucose metabolism, lipid catabolism, and energy metabolism [299]. The activation of TGR5 leads to an increase in cAMP levels, activation of PKA, closure of potassium channels, cell depolarization, influx of calcium ions, and ultimately the secretion of GLP-1. Through the promotion of GLP-1 secretion, TGR5 indirectly enhances insulin sensitivity and glycemic control [300,301]. This mechanism constitutes a critical pathway through which bile acids enhance metabolic processes. FXR modulates the expression of GLP-1 receptors in intestinal tissues [263,302] and interacts with downstream PI3K/AKT via the FXR/GLP-1 axis. GLP-1 augments the TGR5-mediated increase in energy expenditure, and this synergistic interaction contributes to weight management and obesity prevention [195,299]. Bile acids and GLP-1 collaborate synergistically to enhance β-cell function and survival. Furthermore, FXR influences the expression of leptin receptors and modulates leptin sensitivity [303]. Bile acids indirectly regulate leptin secretion by impacting adipose tissue function [204,304]. Additionally, FXR affects the growth hormone axis by modulating the expression of IGF1 [305].

## 6. Interactions Between Bile Acids, Bile Acid Receptors, and the Gut Microbiome

### Effects of Microorganisms on Bile Acid Metabolism

The intestinal microbiome is integral to bile acid metabolism, contributing also to the conversion of bile acids affecting their circulation, composition, and signal transduction. This interaction between microbes and bile acids significantly influences host metabolism and health [17,306]. Bacterial bile acid hydrolase (BSH) facilitates the deconjugation of bile acids [307] and is predominantly produced by Bifidobacterium, Lactobacillus, and Clostridium species [308]. The enzyme 7α-dehydroxylase is responsible for converting primary bile acids into secondary bile acids, such as the transformation of cholic acid (CA) into deoxycholic acid (DCA) and chenodeoxycholic acid (CDCA) into lithocholic acid (LCA) [309]. Microorganisms are also capable of catalyzing the oxidation and reduction of bile acids through enzymes such as 3α-hydroxydehydrogenase and 7α-hydroxydehydrogenase [310]. Additionally, they can catalyze the isomerization of bile acids, exemplified by the formation of ursodeoxycholic acid (UDCA), with contributions from genera such as Bacteroides, Clostridium, Escherichia coli, Eubacterium, Peptostreptococcus, and Ruminococcus [311].

There exists a dynamic interaction between the microbiome and bile acids, wherein bile acids influence the composition of the intestinal microbiome, which in turn modulates the composition and size of the bile acid pool [312]. Microbial metabolism generates a diverse array of atypical bile acids, thereby enhancing the structural diversity of bile acids [6,9,313]. Intestinal microorganisms enzymatically convert bile acids synthesized in the liver into secondary bile acids, such as chenodeoxycholic acid (CDCA), deoxycholic acid (DCA), and lithocholic acid (LCA), which can act as natural ligands for the FXR. Microbial activity influences the extent of FXR activation in both the intestine and liver [314]. Furthermore, products of microbial metabolism, including secondary bile acids such as LCA and DCA, serve as potent agonists of the TGR5, thereby impacting TGR5-mediated glucagon-like peptide-1 (GLP-1) secretion and energy metabolism [175,297,315]. Microbial-mediated conversion of bile acids plays a critical role in regulating glucose homeostasis, lipid metabolism, and energy balance through the activation of FXR and TGR5 signaling pathways [298,316]. Bile acids are not only vital for the digestion of fats but also function as signaling molecules that modulate various metabolic processes. Research indicates that the composition and functionality of the intestinal microbiota significantly influence bile acid metabolism, which subsequently affects the metabolic status of the host [317]. By activating FXR and TGR5 receptors in the liver and intestine, bile acids enhance the metabolism of glucose and lipids, thereby contributing to the regulation of systemic energy balance [264].

Under conditions of a high-fat diet, alterations in the composition and concentration of bile acids may contribute to metabolic disorders, including obesity and type 2 diabetes [318]. Modifications in the gut microbiota can influence insulin sensitivity and lipid metabolism by affecting the synthesis and conversion of bile acids, thereby impacting the activation states of the nuclear receptor FXR and the G-protein-coupled receptor TGR5 [319]. For instance, certain microorganisms possess the ability to modify bile acid structures through the action of bile salt hydrolase (BSH), enhancing their efficacy in activating FXR and TGR5, which in turn can improve glucose tolerance and lower blood lipid levels [302,320]. Furthermore, bile acid metabolites may exert regulatory effects on the host’s metabolic status by modulating the composition of the intestinal microbiota [18,321]. For instance, certain dietary components, such as polyphenols, have the potential to enhance metabolic health by altering the structure of the intestinal microbiota and facilitating the production of beneficial bile acids [322,323]. Consequently, microbial-mediated bile acid transformation influences the biological activity of bile acids also plays a significant role in host metabolism via the FXR and TGR5 signaling pathways. In conclusion, the conversion of bile acids mediated by the microbiota is pivotal in maintaining glucose homeostasis, lipid metabolism, and energy balance through the FXR and TGR5 signaling pathways, offering novel insights and targets for the treatment of metabolic diseases [324].

Microbial-mediated bile acid metabolism plays a crucial role in drug metabolism, encompassing drug activation and toxicity. Bile acids serve as essential components in lipid digestion also as signaling molecules that regulate diverse physiological processes. Research has demonstrated that intestinal microorganisms influence drug metabolic pathways by converting primary bile acids into secondary bile acids [325]. These secondary bile acids modulate the expression of drug-metabolizing enzymes through interactions with nuclear receptors, such as FXR and TGR5, thereby impacting the bioavailability and clearance of drugs [326,327]. Furthermore, bile acids metabolized by microorganisms can lead to drug activation or inactivation. For instance, certain drugs may be transformed by microorganisms into active forms within the intestine, thereby enhancing their efficacy [328]. Nevertheless, microbial metabolism has the potential to generate toxic metabolites, thereby exacerbating drug toxicity [329]. Consequently, it is imperative to comprehend the influence of microorganisms on bile acid metabolism and its subsequent effects on drug metabolism, as this knowledge is vital for advancing personalized medicine and drug design [330]. In the context of drug development, accounting for the effects of microorganisms on bile acid metabolism can enhance predictions of drug efficacy and safety. By incorporating metabolomics and microbiome methodologies, researchers can gain a more profound understanding of the role played by intestinal microorganisms in drug metabolism, thus offering novel insights for the development of new pharmaceutical agents [331].

## 7. Roles of Bile Acids and Bile Acid Receptors in Intestinal Diseases

### 7.1. Roles in Inflammatory Bowel Disease

In individuals with inflammatory bowel disease (IBD), disturbances in bile acid metabolism are characterized by impaired bile acid absorption [332,333], altered bile acid pool composition [334], and abnormal enterohepatic circulation [335].

In the context of IBD, the expression and functionality of the FXR are notably compromised. Some articles have reported that individuals with IBD exhibit diminished FXR activity, potentially linked to dysbiosis of the intestinal microbiota and irregularities in bile acid metabolism [336]. In murine models, the ablation of FXR has been associated with compromised intestinal barrier integrity and heightened intestinal permeability, which in turn exacerbates hepatic steatosis and the inflammatory response [337]. FXR is integral to the modulation of intestinal inflammation, serving a critical anti-inflammatory function. It mitigates intestinal inflammation by suppressing the expression of pro-inflammatory cytokines, including tumor necrosis factor alpha (TNF-α) and interleukin-6 (IL-6) [338]. Furthermore, the activation of FXR has been shown to facilitate the proliferation of beneficial bacteria by modulating the composition of the intestinal microbiota, thereby augmenting the intestine’s anti-inflammatory capacity [339]. The FXR is integral to the maintenance of intestinal barrier function. Activation of FXR enhances the integrity of tight junctions in intestinal epithelial cells, thereby reducing intestinal permeability and preventing the translocation of harmful substances [340]. Empirical evidence suggested that FXR agonists bolster the integrity of these cells and mitigate damage associated with intestinal inflammation [341]. FXR is also involved in bile acid metabolism and hepatic metabolic regulation through its modulation of the intestinal hormone fibroblast growth factor 19 (FGF19). Following FXR activation, FGF19 is secreted as a principal intestinal hormone, which subsequently inhibits CYP7A1, a critical enzyme in hepatic bile acid synthesis, thus preserving bile acid homeostasis [342]. In the context of IBD, FGF19 expression is frequently downregulated, potentially leading to bile acid metabolism dysregulation and exacerbating intestinal inflammation [343]. FXR is pivotal in the pathophysiology of IBD, influencing inflammatory responses, maintaining intestinal barrier integrity, and modulating the FGF19 signaling pathway. Alterations in FXR functionality may therefore be a crucial factor in the disease process of IBD (Table 1).

The anti-inflammatory properties of TGR5 in the context of IBD encompass its roles in modulating intestinal motility, secretion, and immune regulation. Research indicates that TGR5 is integral not only to the regulation of intestinal motility and secretion but also to the modulation of immune responses, thereby influencing the anti-inflammatory outcomes in IBD [70]. Initially, TGR5 contributes to the progression of IBD by modulating intestinal motility. Proper intestinal motility is crucial for maintaining intestinal health, and TGR5 activation facilitates the contraction of intestinal smooth muscle, thereby enhancing intestinal peristalsis. This mechanism aids in the reduction of inflammatory substance accumulation within the intestine, consequently alleviating IBD symptoms [344]. Furthermore, TGR5 exerts anti-inflammatory effects through the promotion of intestinal secretion. Studies have demonstrated that TGR5 activation enhances the secretory function of intestinal epithelial cells, leading to increased release of anti-inflammatory mediators such as glucagon-like peptide-1 (GLP-1) and intestinal barrier proteins. These factors contribute to the repair of damaged intestinal barriers also to the inhibition of inflammatory responses, thereby reducing inflammation levels within the intestine [345]. Furthermore, the role of TGR5 in immune regulation is significant. TGR5 modulates immune cells in the intestine and promotes the generation of regulatory T cells (Treg), thereby enhancing intestinal immune tolerance. This immunomodulatory effect aids in preventing excessive immune responses and reducing the incidence of IBD [346]. Additionally, TGR5 inhibits the production of proinflammatory cytokines, such as tumor necrosis factor α (TNF-α) and interleukin (IL-6), further mitigating the inflammatory response in the intestine [347]. In summary, TGR5 demonstrates its anti-inflammatory potential in IBD by regulating intestinal motility, enhancing intestinal secretion, and modulating immune responses. These findings offer novel insights into IBD treatment, suggesting that future research could further explore the potential of TGR5 as a therapeutic target (Table 1).

In the context of IBD, the interplay between bile acids, microbial communities, and the host is of significant importance. Research has demonstrated that patients with IBD frequently exhibit dysbiosis, characterized by a reduction in secondary bile acids and alterations in short-chain fatty acids (SCFAs) [348]. Secondary bile acids, such as ursodeoxycholic acid (UDCA) and lithocholic acid (LCA), are recognized for their anti-inflammatory properties, and their synthesis is contingent upon the metabolic activities of intestinal microorganisms [349]. Alterations in the composition of the gut microbiota can influence the production of secondary bile acids, potentially exacerbating the pathological conditions associated with IBD [350]. Furthermore, microbial imbalance may result in diminished SCFA production, thereby compromising the integrity of the intestinal barrier and immune function [348].

Bile acids and their receptors exhibit significant potential in the therapeutic management of IBD. Firstly, the activation of the bile acid receptor FXR is regarded as a critical therapeutic approach. FXR is integral in the regulation of bile acid metabolism, lipid metabolism, and inflammatory responses [351]. Studies have shown that FXR agonists can ameliorate intestinal inflammation and enhance intestinal barrier function, thereby mitigating the symptoms associated with IBD [352]. Secondly, interventions targeting TGR5 (Takeda G protein-coupled receptor 5) also demonstrate considerable promise in IBD treatment. TGR5 is implicated in bile acid metabolism also in the regulation of energy balance and immune responses [353]. Activation of TGR5 can potentiate the intestinal anti-inflammatory response and improve the intestinal microenvironment, thereby offering novel therapeutic avenues for patients with IBD [349]. Furthermore, bile acid supplementation has been suggested as a potential therapeutic approach for IBD. Bile acids, functioning as signaling molecules, have the capacity to enhance intestinal health by modulating the gut microbiota and immune responses [18]. Scholars have conducted research indicating that appropriate bile acid supplementation can foster the proliferation of beneficial bacteria while suppressing pathogenic organisms, thereby ameliorating the intestinal microecological balance in patients with IBD [349,354]. Additionally, interventions targeting the microbiome represent a promising area of investigation. The gut microbiome plays a critical role in the pathogenesis of IBD and can influence its progression by modulating bile acid metabolism and signal transduction pathways [355]. Future research may focus on optimizing bile acid metabolism through microbiome interventions, thereby offering novel insights for IBD treatment [356,357]. In brief, bile acids and their receptors exhibit multiple mechanisms of action in the context of IBD treatment, and ongoing research is expected to further elucidate their potential, providing new directions for clinical applications.

### 7.2. Roles in Colon Cancer

The pro-oncogenic role of bile acids in colorectal cancer (CRC) development encompasses several critical mechanisms, including DNA damage and genomic instability, disruption of the balance between cell proliferation and apoptosis, modulation of the inflammatory microenvironment, and compromise of intestinal barrier integrity. Firstly, the accumulation of bile acids is intricately linked to DNA damage. Research indicates that specific bile acids, such as deoxycholic acid (DCA), can induce oxidative stress, resulting in DNA damage and mutations, thereby facilitating colorectal cancer development [358]. Furthermore, elevated concentrations of bile acids can lead to genomic instability within cells, a hallmark of cancer progression [359]. In colorectal cancer cells, persistent exposure to bile acids can result in aberrant cell cycle regulation, thus accelerating tumor progression [360]. Secondly, the role of bile acids is evident in the disruption of the balance between cell proliferation and apoptosis. A high-fat diet results in elevated bile acid levels, creating an environment that promotes the proliferation of intestinal epithelial cells while inhibiting apoptosis, thereby conferring a survival advantage to tumor cells [361]. This imbalance impairs the physiological functions of normal cells, establishing favorable conditions for the growth of cancer cells as well [362]. Additionally, the impact of the inflammatory microenvironment is a crucial aspect of the carcinogenic effects of bile acids. The accumulation of bile acids can activate the inflammatory response in the intestine, leading to a chronic inflammatory state, which is regarded as a significant driving factor in the development of colorectal cancer [363]. Chronic inflammation exacerbates DNA damage by releasing proinflammatory cytokines and reactive oxygen species (ROS), thereby promoting tumorigenesis [364].

In the context of colon cancer research, alterations in the expression of the FXR are closely associated with tumor suppression, anti-inflammatory effects, and metabolic regulation. As a nuclear receptor, FXR exerts multiple protective roles by modulating the metabolism of bile acids, glucose, and lipids, thereby inhibiting the development of hepatic and intestinal tumors [365]. In cases of colon cancer, FXR expression is typically suppressed, a condition that is strongly linked to tumor progression [108,137]. Empirical evidence indicated that FXR activation can impede the advancement of colon cancer by inhibiting the proliferation of intestinal cancer stem cells. For instance, certain bile acid components can antagonize FXR function, resulting in the proliferation and DNA damage of cancer stem cells, whereas selective FXR activation can mitigate this aberrant growth [50]. Furthermore, the FXR plays a significant role in modulating the growth and survival of tumor cells through the regulation of intracellular metabolic pathways. Notably, FXR influences autophagy by inhibiting the mechanistic target of rapamycin complex 1 (mTORC1) signaling pathway, thereby impacting the malignant progression of colon cancer cells [205]. The anti-inflammatory properties of FXR are equally crucial; FXR agonists mitigate inflammatory damage to hepatocytes by inducing the expression of suppressor of cytokine signaling 3 (SOCS3) [366]. In the context of colon cancer, FXR activation suppresses tumor cell proliferation, enhancing intestinal health by modulating the intestinal microbiota and inflammatory response as well [367]. For instance, FXR activation is linked to alterations in the composition of the intestinal microbiota, which may regulate the metabolic state of the intestine by influencing bile acid metabolism, thus inhibiting the development of colon cancer [367,368]. Furthermore, the significance of FXR in metabolic regulation is undeniable. FXR influences the intestinal metabolic environment by modulating lipid metabolism and bile acid synthesis, which plays a crucial role in the onset and progression of colon cancer [369]. For instance, the activation of FXR enhances the expression of antioxidant enzymes and mitigates oxidative damage, thereby suppressing intestinal inflammation and tumorigenesis [370]. Alterations in FXR expression in colon cancer are intricately linked to its tumor-suppressive, anti-inflammatory, and metabolic regulatory effects. Modulating FXR activity could offer novel therapeutic strategies for the treatment of colon cancer (Table 1).

In the context of colon cancer, bile acids exhibit a dual role in the carcinogenic process [371]. Alterations in the expression of TGR5 may significantly influence tumor cell proliferation and survival. Empirical evidence suggested that TGR5 is implicated not only in the regulation of cell proliferation but also in modulating the tumor microenvironment through its effects on immune responses and metabolic pathways. Specifically, the activation of TGR5 may facilitate the proliferation of colon cancer cells while inhibiting apoptosis, thereby enhancing tumor cell survival [72]. Consequently, TGR5 expression is upregulated in certain tumor tissues [372]. Furthermore, the immunomodulatory role of TGR5 in colon cancer warrants attention, as it may impact the immune evasion mechanisms of tumors by regulating immune cell infiltration and activity [373]. For instance, TGR5 expression is closely associated with the composition and functionality of immune cells within the tumor microenvironment, thereby influencing tumor progression and patient prognosis [371,374]. Regarding metabolic regulation, the activation of TGR5 could potentially enhance tumor cell proliferation by influencing energy metabolism and intracellular signaling pathways [375]. In conclusion, alterations in TGR5 expression in colon cancer may impact tumor cell proliferation, survival, immune regulation, and metabolism through various mechanisms, indicating its potential as a therapeutic target (Table 1).

Microbial dysbiosis can significantly influence bile acid metabolism, resulting in the enhanced production of carcinogenic secondary bile acids [17]. Research indicates that bile acids function as surfactants facilitating lipid digestion also play a crucial role in regulating the synthesis of short-chain fatty acids and modulating epigenetic mechanisms through interactions with host metabolic pathways, all of which may contribute to the progression of colorectal cancer [376,377]. Furthermore, alterations in bile acid profiles are intricately linked to the composition of the gut microbiota, with microbial metabolic activities capable of modifying the structure and function of bile acids, thereby impacting the host’s immune responses and metabolic state [378]. In the context of colorectal cancer, specific microbial communities may enhance the production of carcinogenic bile acids, such as deoxycholic acid (DCA) and lithocholic acid (LCA), which have been implicated in the pathogenesis of this malignancy [379]. Concurrently, bile acids regulate the metabolism of short-chain fatty acids, which are vital for maintaining intestinal health and preventing tumorigenesis [16,380]. Understanding the interactions among bile acids, microbial communities, and the host is essential for developing innovative strategies to prevent and treat colorectal cancer (CRC) [381]. The use of FXR agonists is being explored for the chemoprevention of CRC [382]. Modulating bile acid metabolism could form a component of preventive strategies [383] and may exhibit synergistic effects when combined with conventional chemotherapeutic agents [384]. In contrast, TGR5 antagonists have the potential to inhibit TGR5-mediated tumor promotion [385]. It is important to consider the differential roles of TGR5 across various stages and subtypes of CRC [373,386]. Moreover, the targeted regulation of microorganisms involved in bile acid metabolism may influence CRC risk and progression, suggesting that probiotics or specific microbial metabolites could be employed in CRC prevention [387].

### 7.3. Roles in Irritable Bowel Syndrome

In individuals with irritable bowel syndrome (IBS), approximately 25–30% of patients with diarrhea-predominant IBS (IBS-D) experience bile acid malabsorption [388], frequently accompanied by bile acid diarrhea (BAD), a chronic condition resulting from excessive bile acid influx into the colon [389,390]. Abnormal bile acid metabolism not only disrupts normal intestinal function but may also precipitate systemic inflammatory responses and metabolic disorders [9]. In IBS patients, there may be an upregulation of bile acid synthesis as a compensatory mechanism for bile acid deficiency due to malabsorption [391]. Furthermore, alterations in the composition of the bile acid pool can result in an imbalance between primary and secondary bile acids, potentially influencing the intestinal microbiota’s composition [392]. Research has demonstrated that bile acids play a role in regulating metabolism and immune responses through the activation of the FXR and TGR5 receptor in the intestine [9]. In patients with IBS, aberrant bile acid metabolism may lead to dysregulation of the FXR signaling pathway, consequently impacting bile acid synthesis and secretion in the liver [393]. Thus, interventions targeting bile acid metabolism, such as the use of bile acid binders or modulation of the intestinal microbiota, may offer novel therapeutic approaches for IBS [394].

Approximately 25% of patients with diarrhea-predominant IBS (IBS-D) exhibit bile acid diarrhea (BAD), which is associated with excessive bile acid excretion in the intestines [395]. Elevated bile acid levels can lead to increased intestinal fluid secretion, thereby inducing diarrhea [396]. Moreover, bile acid concentrations have been positively correlated with stool consistency and frequency, suggesting that abnormal bile acid metabolism may directly impact the bowel habits of IBS-D patients [397]. Additionally, bile acids are implicated in the pathogenesis of abdominal pain, as they can activate intestinal nerve receptors and affect sensory nerve function, thereby eliciting abdominal discomfort [398]. In patients with IBS, alterations in bile acid metabolism may contribute to heightened intestinal sensitivity, thereby exacerbating symptoms of abdominal pain [397]. Furthermore, dysregulated bile acid metabolism may influence intestinal permeability. Studies have shown that the intestinal barrier function in patients with diarrhea-predominant IBS (IBS-D) is compromised, resulting in increased intestinal permeability [399]. The integrity of the intestinal barrier is closely associated with bile acid alterations; excessive bile acids can inflict damage on intestinal epithelial cells, consequently enhancing intestinal permeability [400].

In the context of IBS, the FXR is integral to the regulation of intestinal barrier function, inflammation, and bile acid metabolism. Firstly, FXR is vital for the maintenance of intestinal barrier integrity. Empirical evidence suggested that the activation of FXR enhances the tight junctions of intestinal epithelial cells, thereby fortifying the intestinal barrier. This function is critical in preventing the translocation of harmful substances within the intestine, a process particularly relevant to IBS patients, where compromised barrier function may precipitate intestinal inflammation and associated discomfort [401]. Secondly, FXR is instrumental in modulating intestinal inflammation. It mitigates inflammatory responses by regulating the expression of genes associated with immune functions. In individuals with IBS, alterations in FXR expression may contribute to heightened inflammatory activity [400]. For instance, activation of the FXR can suppress the release of proinflammatory cytokines, thus mitigating intestinal inflammatory responses [402]. Moreover, FXR plays a crucial role in the regulation of bile acid metabolism. Bile acids are essential for lipid digestion also function as signaling molecules that influence the intestinal microbiota and various metabolic processes. In patients with IBS, bile acid metabolism may be disrupted, and FXR activation can enhance the synthesis and excretion of bile acids, thereby restoring metabolic balance [91,403]. Research has demonstrated that FXR activation can modulate the composition of the intestinal microbiota, consequently impacting bile acid metabolism and intestinal health [18,404] (Table 1).

TGR5 is purported to play a significant role in the pathophysiology of irritable bowel syndrome (IBS). IBS is a prevalent functional gastrointestinal disorder, characterized by symptoms such as abdominal pain, bloating, and altered bowel habits [405]. Research indicates that TGR5 is implicated in the regulation of intestinal motility also in visceral sensitivity and immune response [406]. The involvement of TGR5 in intestinal motility has been the subject of extensive investigation. Activation of TGR5 has been shown to facilitate the contraction of intestinal smooth muscle, thereby enhancing intestinal motility [407]. This mechanism may hold substantial clinical relevance for patients with IBS, particularly for those with constipation-predominant IBS (IBS-C), as these individuals frequently exhibit symptoms associated with intestinal hypomotility [408]. Secondly, TGR5 is intricately associated with visceral sensitivity, which denotes the capacity to perceive stimulation of visceral organs, such as the intestines. Patients with IBS frequently exhibit heightened visceral sensitivity [409]. Research indicates that the activation of TGR5 can influence visceral sensitivity by modulating neuronal excitability, thereby potentially mitigating abdominal pain and discomfort in IBS patients [410]. Furthermore, the significance of TGR5 in immune regulation warrants attention. The intestinal immune response is pivotal in the pathogenesis of IBS, and TGR5 activation can facilitate the release of anti-inflammatory cytokines, thus suppressing the intestinal inflammatory response [411]. This mechanism may hold substantial therapeutic promise for IBS patients, particularly those experiencing concurrent intestinal inflammation. To sum up, TGR5 is implicated in various aspects of IBS pathophysiology, including the regulation of intestinal motility, visceral sensitivity, and immune response. Future research may further elucidate the potential of TGR5 as a therapeutic target for IBS, offering novel strategies to enhance patient quality of life (Table 1).

The interplay between bile acids, microbial communities, and the host is crucial in the investigation of IBS. Dysbiosis is regarded as a pivotal factor in IBS, with alterations in the diversity and composition of the gut microbiota potentially influencing bile acid metabolism and function [9]. Bile acids are integral not only to lipid digestion but also act as signaling molecules that modulate host metabolic processes and immune responses [355]. Research has demonstrated that secondary bile acids are crucial in modulating the intestinal microbiota and host metabolism, particularly in sustaining intestinal health and preventing inflammation [412]. Furthermore, short-chain fatty acids (SCFAs), which are also byproducts of intestinal microbial metabolism, exert a considerable influence on the host’s metabolic and immune functions. SCFAs may contribute to the pathophysiology of IBS by enhancing intestinal barrier integrity and modulating immune responses [10,413]. Consequently, elucidating the interactions among bile acids, microbes, and the host, particularly in the context of IBS, could inform novel therapeutic strategies. Modulating intestinal microbiota and bile acid metabolism may ameliorate IBS symptoms and inspire innovative treatment approaches [348,414].

**Table 1:** The Function of FXR and TGR5 in Traditional Intestinal Disorders. (The arrows: “↓” and “↑” represent the expression levels of bile acid receptor proteins, respectively).

Disease Type	Bile Acid Receptor	Receptor Function	Mechanism	References
IBD	FXR ↓	FXR regulates inflammatory response, maintains intestinal barrier function, and affects FGF19 signaling pathway.	Dysbiosis of the gut microbiota and aberrant bile acid metabolism are interconnected phenomena.	[336,337,338,339,340,341,342,343]
	TGR5 ↓	TGR5 mitigates the accumulation of pro-inflammatory substances within the intestine. Activation of TGR5 can enhance the secretory functions of intestinal epithelial cells and promotes the release of anti-inflammatory mediators.	Diminished expression of TGR5 is associated with disruptions in intestinal motility, secretion, and immune regulation.	[70,344,345,346,347]
CRC	FXR ↓	Alterations in FXR expression are posited to be intricately associated with tumor suppressive effects, anti-inflammatory responses, and metabolic regulation.	A reduction in FXR expression levels diminishes its inhibitory impact on the proliferation of intestinal cancer stem cells, thereby facilitating the progression of colon cancer.	[50,108,137,366,367,368,369,370]
	TGR5 ↑	TGR5 is involved in the regulation of cell proliferation and may also influence the tumor microenvironment by modulating immune responses and metabolic pathways.	The activation of TGR5 has the potential to facilitate the proliferation of colon cancer cells while simultaneously inhibiting apoptosis, thereby augmenting the tumor’s survival capacity.	[371,372,373,374,375]
IBS	FXR ↓	Activation of the farnesoid X receptor (FXR) has been shown to strengthen the tight junctions of intestinal epithelial cells, consequently enhancing the integrity of the intestinal barrier.	In patients with IBS, the expression of FXR is altered, resulting in the exacerbation of inflammatory processes.	[18,91,401,402,403,404]
	TGR5 ↓	TGR5 is involved in the regulation of intestinal motility and also contributes to visceral sensitivity and immune response.	In patients with IBS, there is a downregulation of TGR5 expression levels, which is associated with diminished intestinal peristalsis, decreased neuronal excitability, altered visceral sensitivity, and a reduced release of anti-inflammatory factors.	[405,406,407,408,409,410,411]

## 8. Conclusions

Over the past few decades, the field of bile acid receptor research has experienced significant advancements, transitioning from the initial identification of pivotal receptors such as FXR and TGR5 to a more comprehensive understanding of their intricate roles in metabolic regulation, inflammatory responses, and cellular signaling. These investigations underscore the significance of bile acids as signaling molecules as well as pave the way for novel therapeutic approaches for various metabolic, hepatobiliary, and inflammatory bowel diseases. Nevertheless, as research progresses, we encounter increasing challenges and opportunities. The development of effective and safe therapies based on bile acid signaling necessitates further basic and translational research to address several critical issues. Firstly, there is a need to bridge the gap between experimental data and clinical application, considering the substantial differences in bile acid composition and intestinal microbiota between humans and animal models. Secondly, it is essential to account for individual variations in intestinal microbiota and investigate how these microbial factors differentially impact disease states and responses to bile acid signaling therapies. Third, it is essential to consider the phasic effects of varying bile acid concentrations on the body. Given the reciprocal interaction between bile acids and microbial communities, it is imperative to elucidate how microbial populations regulate bile acid signaling and, conversely, how bile acid signaling influences gut microbial composition, health, and function. Understanding these dynamic interactions is crucial for designing effective therapeutic strategies.

The intricate cell-specific and spatiotemporal regulation of bile acid receptor function underscores the necessity for comprehensive research. To achieve this, it is vital to develop advanced research tools and methodologies, such as single-cell technologies, high-resolution imaging, and integrated multi-omics analyses, to thoroughly investigate the dynamic alterations in the bile acid signaling network across various physiological and pathological contexts. Grasping this complexity is fundamental for the development of more precise therapeutic interventions. The pivotal role of the gut microbiome in bile acid metabolism and signaling is widely acknowledged. Future research should aim to further elucidate the molecular mechanisms underlying microbial–bile acid–host interactions and investigate strategies to enhance health by modulating this axis. Such endeavors may pave the way for innovative microbiome-based interventions, serving as either supplements or alternatives to conventional pharmacological treatments. The design of highly specific ligands for receptors such as FXR and TGR5 remains a complex and challenging area of study. However, recent advancements in structural biology, computational chemistry, and artificial intelligence have introduced novel tools for ligand design. Future investigations should prioritize the development of new ligands characterized by tissue selectivity and regulatable activity, as well as the exploration of bifunctional or multifunctional ligands. These efforts are anticipated to yield more efficacious therapeutic agents with reduced side effects. Furthermore, as our comprehension of interindividual variations in bile acid signaling pathways deepens, the formulation of personalized treatment strategies becomes increasingly imperative. This encompasses the development of precise diagnostic and classification tools, the design of personalized dosing regimens, and the utilization of artificial intelligence and digital twin technology to forecast treatment outcomes. Attaining this objective necessitates interdisciplinary collaboration that synthesizes the strengths of fundamental research, clinical medicine, and data science.

## Data Availability

Data sharing is not applicable to this article as no datasets were generated or analyzed during the current study.

## Abbreviations

The following abbreviations are used in this manuscript:

FXR	the farnesoid X receptor
TGR5	G protein-coupled bile acid receptor
IBD	inflammatory bowel disease
CRC	colorectal cancer
IBS	irritable bowel syndrome
GLP-1	glucagon-like peptide-1
TLRs	Toll-like receptors
NLRs	NOD-like receptors
EGFR	epidermal growth factor receptor
ROS	reactive oxygen species
IEC	intestinal epithelial cell
MCU	mitochondrial calcium uniporter
BSH	bacterial bile acid hydrolase
